# Annotations

**Published:** 1894-10-27

**Authors:** 


### Annotations.

### Barracks or Villages for Children?

The controversy which has for some time been carried on in the columns of the *Times* and elsewhere as to the relative merits of the barrack and the boarding-out system for pauper children has been ably dealt with in a leader in that journal. The *Times* gives the verdict in favour of boarding out; and no one who has any practical knowledge of the working of the two systems can doubt that the leading journal is right. From the point of view of bodily vigour, to say nothing of freedom from infectious diseases, there is no comparison between the two systems; the merits are all on the side of boarding out. Similarly from the standpoint of a "start in life" for the boys and girls the village would seem to be much to be preferred to the barrack. For the barrack, let it be remembered, is generally an isolated institution having no connection with any town or even village, but only with the parish authorities who maintain it. The village gives the boys the opportunities of farm labour, of service in the families of the neighbouring gentry, of work in market gardens, of apprenticeship to local tradesmen, of the merchant service at sea, and so on. These are exactly the employments suited for poor and friendless children, and, moreover, they are the employments where the least competition for vacancies will be found. The objections to the village system are that inspection is perfunctory, and that the children are, or may be, farmed. This is said to be actually the case at Eyke, in Suffolk. There is, we believe, one ready and perfect remedy against "farming" and perfunctory inspection. Make the village doctor the salaried inspector, and he will do all that is necessary. "Farming" and neglect will alike be impossible under his trained and watchful eye.

### Anti-Cholera Inoculation in India.

The experiments now being made in India by M. Haffkine, of the Pasteur Institute, and which we have from time to time discussed with our readers from their commencement, have become the subjects of keen controversy in medical and military circles in our great dependency. A few months ago we announced, on authority of Dr. Simpson, that some remarkable
the authority of Dr. Simpson, that some remarkable
results had been obtained in certain bustees near Calcutta, results which pointed hopefully in the direction of the complete success of M. Haffkine's anti-cholera inoculations. More recent information, which we have received from both public and private sources, indicates, unhappily, some conclusions of a very different character. It is matter of common knowledge that the East Lancashire Regiment at Lucknow has just been passing through a cholera epidemic of an exceptionally severe character. In that single regiment no fewer than 140 were attacked, and the terrible total of 95 died. Haffkine's inoculations were obviously indicated at the commencement of the outbreak, by way of prophylaxis, and 130 healthy men of the regiment were accordingly inoculated. It must be admitted that this, though not an unfair, was a very severe test of the value of the new method. Unfortunately, so far at any rate as this particular experiment is concerned, the inoculation has quite failed to justify itself. Of the 130 men inoculated 17 were attacked and 12 died. In the whole regiment 140 were attacked and 95 died. In other words, the proportion of inoculated persons attacked, and the proportion of deaths among them, were practically identical with the proportion of uninoculated attacked and the proportion of deaths among them. So that, neither as a preventive of cholera attacks, nor as a modifier of severity in those seized, does the Haffkine inoculation seem to possess any virtue. Scientific persons, however, will be very unwilling to condemn the inoculations in consequence of the failure of merely one experiment, exactly as they were unwilling to jump to assured conclusions on the strength of one or two apparently successful trials of the treatment. The truth is that inoculative prophylaxis as a whole is now on its trial. It is in no sense a line of treatment which lends itself to hurried conclusions. On the contrary, it will take many years in point of time, innumerable experiments by all sorts and conditions of experimenters in all parts of the world, and the most careful philosophic reasoning on the part of competent thinkers as well, before medicine as a whole will be able to pronounce upon its value. "Raw haste," to use Tennyson's phrase, is the very antipodes and opprobrium of all truly scientific work. All persons who follow science have sooner or later to learn to "possess their souls in patience."

### The Czar's Kidney Affection.

The fact that so exalted a person as the Czar of all the Russias has been attacked by a distressing and, in all probability, mortal affection of the kidneys in the full vigour of middle life, has directed anew the attention of the public to the exceedingly grave character of the chronic diseases of those organs. So far as can be ascertained, the Emperor is suffering from cirrhosis of the kidney, of which name other synonyms are "gouty kidney," "granular contracting kidney," and "small red kidney." This in its full development is a serious affection, and in advanced conditions a hopeless one, for it involves the extensive destruction of the parenchyma, or true secreting and excreting substance of the organ. Having its cause in some hitherto ill-recognized perversion of metabolism in the tissues and the blood, it attacks successively the glomerular tufts of the kidney, the convoluted tubules, or secreting elements, and later the straight tubules, which bear away the secretions and excretions to the ureters and the bladder. Most of all is the connective tissue or framework of the organ affected, and this increases to such an extent that pressure of a destructive character is made upon the essential kidney elements. The net result is a steadily increasing disintegration and destruction of those elements; and when that destruction has proceeded to a sufficient length, there is no longer any secreting capacity in the organ, and death from uræmia or other cause is inevitable. An affection like this, in its advanced stages, presents, as will be seen, a problem to medicine which is, at any rate in a curative sense, insoluble. The only hope for persons suffering from cirrhotic kidney lies in the arrest of the disease by sound treatment in the earlier stages of the disease.

